# Scutellarin Mitigates Aβ-Induced Neurotoxicity and Improves Behavior Impairments in AD Mice

**DOI:** 10.3390/molecules23040869

**Published:** 2018-04-10

**Authors:** Yue-Qin Zeng, Yin-Bo Cui, Juan-Hua Gu, Chen Liang, Xin-Fu Zhou

**Affiliations:** 1 Key Laboratory of Stem Cells and Regenerative Medicine, Institute of Molecular and Clinical Medicine, Kunming Medical University, Kunming 650500, China; gujuanhua86@163.com (J.-H.G.); riverjuly@163.com (C.L.); 2 Department of Biochemistry, College of Basic Medicine, Kunming Medical University, Kunming 650500, China; cherry172010@163.com; 3 School of Pharmacy and Medical Sciences, Sansom Institute, University of South Australia, Adelaide 5001, SA, Australia

**Keywords:** Alzheimer’s disease (AD), scutellarin, Aβ-amyloid neurotoxicity, APP/PS1 transgenic mice

## Abstract

Alzheimer’s disease (AD) is pathologically characterized by excessive accumulation of amyloid-beta (Aβ) within extracellular spaces of the brain. Aggregation of Aβ has been shown to trigger oxidative stress, inflammation, and neurotoxicity resulting in cognitive dysfunction. In this study, we use models of cerebral Aβ amyloidosis to investigate anti-amyloidogenic effects of scutellarin in vitro and in vivo. Our results show that scutellarin, through binding to Aβ42, efficiently inhibits oligomerization as well as fibril formation and reduces Aβ oligomer-induced neuronal toxicity in cell line SH-SY5Y. After nine months of treatment in APP/PS1 double-transgenic mice, scutellarin significantly improves behavior, reduces soluble and insoluble Aβ levels in the brain and plasma, decreases Aβ plaque associated gliosis and levels of proinflammatory cytokines TNF-α and IL-6, attenuates neuroinflammation, displays anti-amyloidogenic effects, and highlights the beneficial effects of intervention on development or progression of AD-like neuropathology.

## 1. Introduction

Alzheimer diseases (AD) is the most common kind of neurodegeneration disease in the elderly, characterized by deterioration of cognitive functions, extracellular Aβ deposits and intracellular neurofibrillary tangles, other pathology features include the cholinergic neurons and synaptic degeneration/loss, cerebral amyloid angiopathy (CAA), neuroinflammation, and oxidative damage [1,2].

AD is so chronic and complex disease. To date, there is no current disease-modifying therapy available for the treatment of this disorder. In spite of extensive academic, pharmaceutical, and medicinal research, numerous drug candidates targeting Aβ or β-secretase have failed in clinical trials. This means drugs with single targets have less therapeutic effects or cause side effects when used to prevent or treat AD. Therefore, the new strategy towards development of safe agents with multiple targets, natural polyphenols are likely to comply with these requirements due to the advantages of multi-target effects and fewer side effects.

Flavonoids are the largest group of polyphenols available from dietary fruits and vegetables and have been found to play a neuroprotective role by inhibiting or modifying the self-assembly of the amyloid-β (Aβ) peptide into oligomers and fibrils, which are linked to the pathogenesis of Alzheimer’s disease [3]. Scutellarin is a flavonoid found in the traditional Chinese herbal medicine Erigeron breviscapus (vant.), and has been widely used in clinical to treat cardiovascular diseases and cerebrovascular injury [4]). Recently, scutellarin has been proven to: promote neuroprotective effects by inhibition of microglia inflammatory activation [5], attenuate the neurotoxicity of Aβ and protects against Aβ-induced learning and memory deficits in rats [6], exert potential neuroprotective effects on AD. However, up until now the long term multiple antiamyloidogenic and fibril-destabilizing effects have not been studied. In this research, we report that scutellarin efficiently inhibits oligomerization and fibril formation through binding to Aβ42. In the meantime, scutellarin mitigates amyloid pathology and related cognitive deficits after nine months of treatment in APP/PS1 transgenic mice. This research reveals scutellarin is a potential multi-targeting agent against AD.

## 2. Materials and Methods

### 2.1. Aβ Disaggregation Assay

#### 2.1.1. Aβ Preparation

Synthetic Aβ42 peptide corresponding to the human sequence was purchased from Tocris Bioscience, which was dissolved in HFIP at a concentration of 1 mg/mL and was separated into aliquots in sterile Eppendorf Tube (100 μg/tube), followed by incubation at room temperature for 24 h in the fume hood to form clear peptide film, the resulting peptide films were dried under vacuum overnight and stored at −20 °C before assaying [7].

#### 2.1.2. Thioflavin T Fluorescence Assay

**Figure 1:**
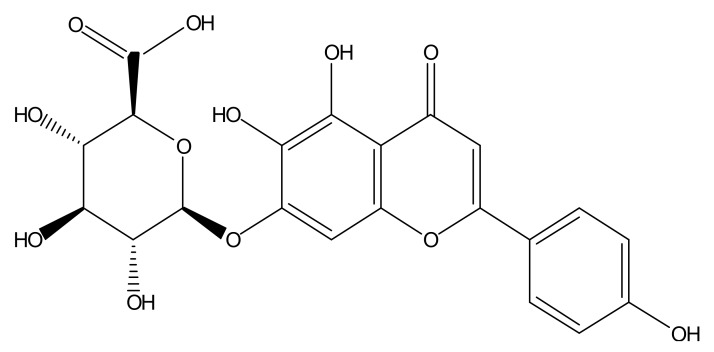
Molecular structure of scutellarin.

Thioflavin T (Th. T) dye fluorescence is used regularly to quantify the formation and inhibition of amyloid fibrils in the presence of anti-amyloidogenic compounds such as polyphenols [8]. To investigate the disaggregation effect of scutellarin on preformed Aβ fibril, 100 μg Aβ42 oligomer was pre-incubated in DMEM at 37 °C for 10 days to form Aβ fibril. Following incubation, Aβ alone, or in the presence of the concentration gradient of scutellarin (purity > 98.5%, Yunnan Plant Pharmaceutical, Yunnan, China, Figure 1) for an additional three days at 37 °C, 5 μM Thioflavin T solution was added to the samples. Emission spectra were recorded at 482 nm upon excitation at 450 nm (SpectraMax M2, Molecular Devices). Measurement was repeated three times.

#### 2.1.3. Electron Microscopy (EM) Assay

In brief, copper grids were preplaced on the bottom of wells in a 24-well plate where 100 μg Aβ42 monomer were incubated with or without scutellarin at a concentration gradient for seven days at 37 °C. Following, a drop of the solution was placed on a 150 mesh Formvar coated grid for 30 s. After drying, the grid was stained with 2% (wt/vol) aqueous phosphotungstic acid for 20 min. The observations were performed on a Hitachi7650 TEM equipped with MegaView 3 Digital Camera (Hitachi, Tokyo, Japan).

### 2.2. SH-SY5Y Cell Culture and Viability Assay

The SH-SY5Y cell line was obtained from BeNa Culture Collection (Shanghai, China). Cells were plated in 96-well plates containing complete medium and cultured for 24 h at 37 °C and then, the cells were treated with 1 μM Aβ42, Scutellarin (2.5, 5, or 10 μM) or 1 μM Aβ42 with Scutellarin (2.5, 5, or 10 μM) for another 24 h, followed by incubation with MTT (0.5 mg/mL) for 4 h and 10% SDS solution for another 15 min at 37 °C. The absorbance was measured at 560 nm with a microplate reader (Spectra Max M2, Molecular Devices, San Jose, CA, USA).

### 2.3. Animals

APP/PS1 transgenic mice and age-matching non-transgenic (WT) mice were purchased from Nanjing Biomedical Research Institute of Nanjing University and were bred in the Kunming Medical University animal house. Five or six mice were housed in each cage with free access to standard food and water and were maintained under standard laboratory conditions. The APP/PS1 transgenic mice were constructed on a C57BL/6 background and bear a chimeric mouse/human (Mo/Hu) APP695 with mutations linked to familial AD (KM 593/594 NL) and human PS1 carrying the exon-9-deleted variant associated with familial AD (PS1dE9) in one locus under control of a brain- and neuron-specific murine Thy-1 promoter element [9]. Genotypes of the offspring were determined by PCR analysis of tail DNA.

### 2.4. Diet Treatment

A total of 24 transgenic mice and 12 wild type littermates (C57BL/6 mice, half male and half female) were used: the transgenic mice were randomly divided into Scutellarin Tg group (N = 12, half males and half females), control Tg group (N = 12, half males and half females) and wild-type mice group (N = 12, half males and half females). The scutellarin (Tg) group were treated with scutellarin mixed food (50 mg/kg) [10]. Control (Tg) and wild-type mice group were fed with standard commercial food (Beijing KeaoXieli Feed Company, Beijing, China). Beginning at three months of age, the mice consumed both diets ad libitum for nine months. This study was carried out in strict compliance with the Guidelines for the Animal Care and Use of China. The protocols were approved by the Animal Ethics Committee of Kunming Medical University.

### 2.5. Behavioral Procedures

The effect of scutellarin treatment on spatial learning and memory was assessed by Morris water maze (MWM) testing at the age of 12 months, a circular plastic pool (height 40 cm, diameter 120 cm) was filled with water (plus white dye) maintained at 22 ± 1 °C. An escape platform (11 cm in diameter) 1 cm below the water surface was used. The acquisition task consisted of six consecutive days of testing with four trials per day. In each trial, the animal was put into the water at one of four starting positions of non-platform quadrants respectively, and was given 60 s to find the hidden platform. If a mouse failed to find the platform within 60 s, the training was terminated, a maximum score of 60 s was assigned, and the mouse was manually guided to the hidden platform. The escape latency, path length and swim speed were recorded semi-automatically by a video tracking system (Stoelting Co., Wood Dale, IL, USA) and analyzed by image analyzing software. On the sixth day, a probe trial was performed, and mice were placed and released opposite the site where the platform had been located, the time spent in each quadrant and target annulus crossings were recorded [11].

### 2.6. AD Type Pathology and Bioanalysis

#### 2.6.1. Tissue Processing

The mice were executed after behavioral test, the mice were deeply anesthetized, the blood was collected from the retro orbital sinus and was centrifuged immediately at 1300× *g* for 8 min, the supernatant from these samples was collected for future biochemical detection.

The brain was perfused intracardially with chilled 0.1 M phosphate buffered saline (PBS) through the heart using a syringe and was quickly removed and placed on an ice-cold glass dish and bisected sagittally. The left-hemisphere were routinely fixed in 4% paraformaldehyde (pH 7.4) for 24 h and incubated in 30% sucrose at 4 °C for 36 h, the coronal sections were cut on a frozen microtome in 35 μm thick sections for staining. The right hemisphere was rapidly frozen in liquid nitrogen and kept at −80 °C until analysis.

#### 2.6.2. Histological Staining and Imagine Analysis

The basic immunohistochemical staining was carried out following IHC kit instruction (Slide Kit Chemical international, Inc., Millipore, Burlington, MA, USA). Five sections each 210 μm apart starting from septal hippocampus were randomly selected and stained with Aβ protein deposits (Biotin-conjugated mouse anti-Aβ antibody (6E10, Serotec, Oxfordshire, UK), activated microglia (rat monoclonal anti-CD45 Chemicon, Temecula, CA, USA), and astrocyte (rabbit poly-clonal anti-glial fibrillary acidic protein, Dako, Glostrup, Denmark)). Pretreatments were: incubation in a 3% H_2_O_2_ solution in distilled water for 20 min to block endogenous peroxidase; incubation in 70% formic acid for 15 min for antigen retrieval, sections were incubated with the primary antibodies overnight at +4 °C, followed by incubation with biotinylated secondary antibodies and visualization using the diaminobenzidine as chromogen. The reaction was stopped with phosphate buffer and sections were then mounted, cleared in xylene, and cover slipped with neutralized resin.

The region of neocortex and hippocampus manually was selected for analysis Aβ, microgliosis and astrocytosis burden. Stained specimens were analyzed with Olympus BX-51 microscope equipped with an Olympus DP-73 camera (Olympus, Tokyo, Japan). Images were collected at 4× magnification. Measurements were performed for a percentage of the area covered by the DAB staining.

#### 2.6.3. ELISA Assay for Soluble and Insoluble Aβ

The levels of soluble and insoluble Aβ in the brain of APP/PS1 mice were quantified according to the procedures previously described [12]. Frozen brain was homogenized and sonicated in water containing 2% sodium dodecyl sulphate (SDS) and protease inhibitors (Roche, Basel, Switzerland). Homogenates were centrifuged at 100,000× *g* for 1 h at 4 °C, the resultant supernatant was collected, representing the TBS-soluble fraction (Aβ-TBS). The resultant pellet was suspended and sonicated in water containing 2% sodium dodecyl sulfate (SDS) and centrifuged at 100,000× *g* for 1 h at 4 °C, the resultant supernatant was collected, representing the SDS-soluble fraction (Aβ-SDS). The resultant pellet was extracted with 70% formic acid (FA) and the supernatant was collected, representing the SDS-insoluble fraction (Aβ-FA). Before ELISA assay, formic acid extracts were neutralized by 1:20 dilution into 1 M Trisphosphate buffer, pH 11, and then diluted in sample buffer. Then, ELISA kits were used to determine Aβ40 and Aβ42 levels in the brain and serum samples (Millipore, Burlington, MA, USA). The concentration of Aβ was expressed as picograms per milliliter (pg/mL). The concentration values of the samples fell within the linear section of the standard curve.

#### 2.6.4. Quantification of Inflammatory Cytokines in the Mouse Plasma by ELISA 

Plasma cytokine levels were measured using commercial assay kits according to the manufacturer’s directions (Thermo Scientific, Waltham, MA, USA). The concentration of inflammatory cytokines was presented as pg/mL.

### 2.7. Statistical Analysis

Results for experimental groups are presented as the mean ± SEM. In cases of equal variance, statistical differences were determined using one-way analysis of variance (ANOVA) followed by post-hoc (Tukey’s) tests for comparisons between groups. If homogeneity of variances was rejected, the ANOVA followed with the Dunnett’s test. The threshold for statistical significance was set to *p* < 0.05. All statistical analyses were performed using the SPSS 20.0 software (IBM, Chicago, IL, USA)

## 3. Results

### 3.1. Protective Effect of Scutellarin on the Cytotoxicity of Aβ42

**Figure 2:**
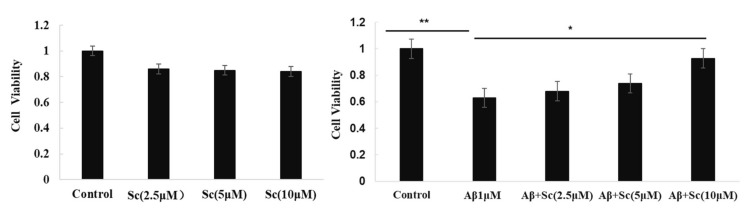
Scutellarin alleviates the toxic effect of Aβ42 in SH-SY5Y cells. MTT assay of cell viability of SH-SY5Y cells treated with (**A**) various doses of Scutellarin for 24 h or (**B**) 1 μM Aβ42 with various doses of Scutellarin (F = 129.553, *p* < 0.01, * *p* < 0.05, ** *p* < 0.01).

Some research conclusions indicate that scutellarin is an effective compound for the prevention of AD-like neuropathology. Here, we used MTT assay to test if scutellarin can reduce cytotoxicity induced by Aβ42 in human neuroblastoma cell line (SH-SY5Y). Our results indicated the Aβ-neurotoxicity in the presence of scutellarin tended to be lower than Aβ in the absence of the drug, scutellarin at 10 uM exhibited significant protective effect against Aβ-induced cytotoxicity (*p* < 0.05, Figure 2). Furthermore, cell viability did not decrease after exposure to different concentrations Scutellarin (2.5, 5, 10 uM), which suggests a good safety profile (Figure 2A).

### 3.2. Effects of the Scutellarin On Aβ42 Oligomerization and Fibrillation

**Figure 3:**
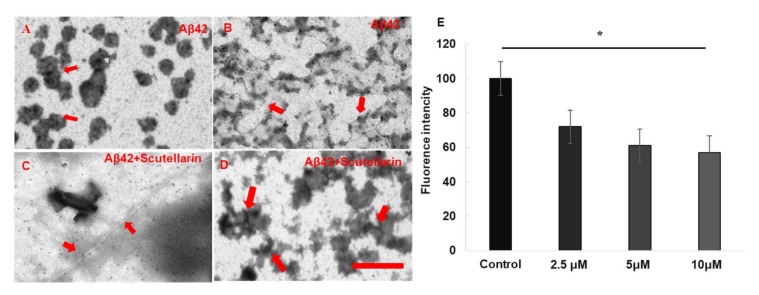
EM images and quantitative analyses Aβ formation in the presence or absence of scutellarin. (**A**,**B**) Aβ42 was incubated alone at 37 °C. The red arrows in (**A**) indicate the oligomers. The red arrows in (**B**) indicate the prefibrillars; (**C**,**D**) Scutellarin was incubated with Aβ42 aggregates (1:5) at 37 °C for an additional 3 d. Scutellarin promoted conversion of Aβ42 to amyloid fibrils (**C**) and disturbed fibril structure and induced the formation of scutellarin-bound amorphous aggregates (**D**). The arrows in (**C**) indicate the fibrils and the arrows in (**D**) indicate amorphous aggregates. Scale bar, 500 nm. (**E**) Th. T fluorescence assays for effect of Scutellarin disaggregation of preformed Aβ fibrils (*n* = 3 per assay, * *p* < 0.05).

Thioflavin T (Th. T) dye fluorescence is used regularly to quantify the formation and inhibition of amyloid fibrils in the presence of anti-amyloidogenic compounds such as polyphenols [8]. Here, we used this dyeing method to detect whether scutellarin promotes the disaggregation of preformed Aβ fibrils. We have found that when scutellarin was incubated with preformed Aβ42 fibrils, the fluorescence intensity of preformed Aβ42 fibrils was dose-dependently reduced (F = 157.16, *p* < 0.05, Figure 3E). Moreover, transmission electron microscopy (TEM) assays visually confirmed that scutellarin promotes amyloid fibril conversion by reducing the pre-fibrillar/oligomeric species of Aβ, resulting in a reduced neurotoxicity induced by Aβ (Figure 3A–D).

### 3.3. Scutellarin Improves Behavioral Impairment after Nine Months of Treatment

**Figure 4:**
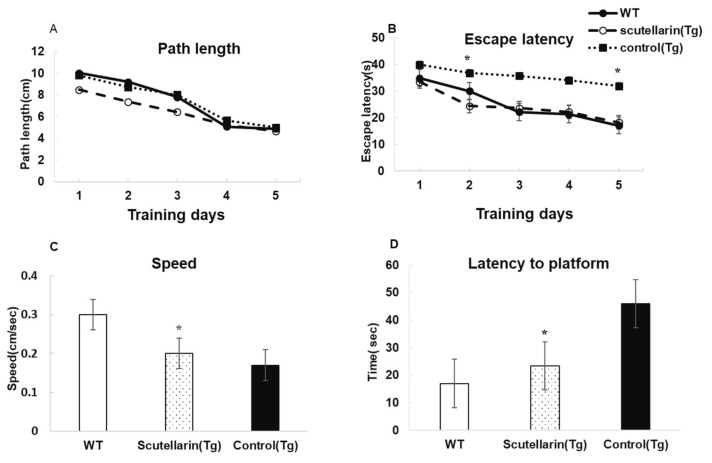
Scutellarin improves behavioral performances of APP/PS1 mice on spatial memory measures in MWM. (**A**) No significant differences are observed in path length among the groups (* *p* > 0.05); (**B**) Significant differences in escape latency among the groups (ANOVA with Bonferroni post hoc * *p* < 0.05); (**C**) WT control group swam significantly faster than other two groups (ANOVA with Dunnett’s post hoc: * *p* < 0.05); (**D**) Scutellarin reduced the latency to find platform in the probe test (ANOVA with Bonferroni post hoc * *p* < 0.05 vs. APP/PS1 control mice, *p* * > 0.05 vs. WT control mice).

Morris water maze is a widely used tool to assess the spatial learning and memory capacities. After nine months of treatment, we evaluated the preventive effects of scutellarin on cognitive deficits in APP/PS1 mice. The results of the behavioral performance are shown in Figure 4. Overall, a time-dependent acquisition of platform location was observed in all groups, as evident in the reduced latency to find the platform with each successive training day in all groups (ANOVA F = 7.518, *p* < 0.05; Figure 4A). In addition, a significant effect of the treatment group was observed (*p* < 0.05). The scutellarin-treated APP/PS1 mice showed significantly shorter latencies than Tg control mice on day 2 (*p* < 0.05) and day 5 of training (*p* < 0.05, Figure 4B). Treatment with scutellarin at 50 mg/kg significantly reduced the latency to find platform (ANOVA F = 6.860, *p* < 0.05, Figure 4D). The effect could not be attributed to a change in swim speed because all APP/PS1 mice swam slower than the wt control group during the probe trial regardless of treatment (Dunnett’s post hoc APP/PS1 vs. wt: *p* < 0.05; Figure 4C)

### 3.4. Scutellarin Reduces Aβ Burden in APP/PS1 Mice

**Figure 5:**
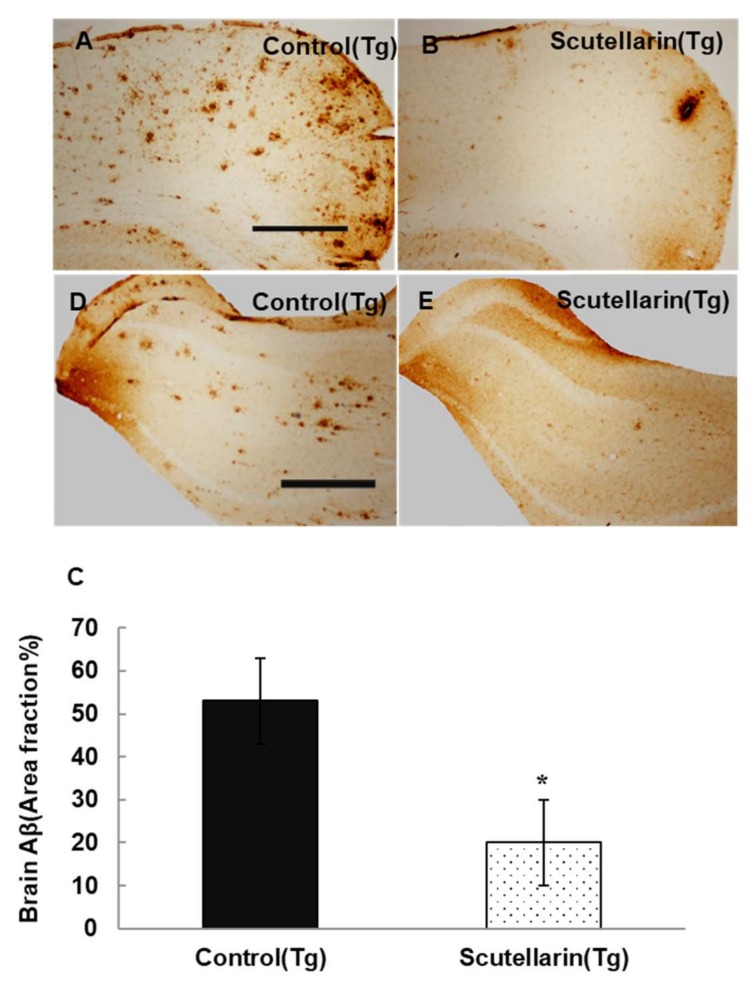
Cutellarin reduced Aβ burden in the scutellarin treated APP/PS1 mice. (**A**–**D**) Aβ plaques in cortex and hippocampus of control transgenic mice; (**B**–**E**) APP/PS1 transgenic mice treated with scutellarin diet; (**C**) Comparison of Aβ plaque area fraction in neocortex and hippocampus between groups. * *p* < 0.05 versus APP/PS1 control mice. Scale bar = 0.5 mm. Original magnification, 4×.

In order to study the histopathological changes after the treatment, the Aβ plaques in the brain were stained with immunohistochemical (IHC) staining (biotin conjugated mouse anti-Aβ antibody 6E10, Serotec, USA). The statistical results show APP/PS1 mice treated with scutellarin significantly decreased 63% Aβ deposits in the brain compared to the Tg Control group (ANOVA F = 12.105, *p* < 0.001) (Figure 5).

**Figure 6:**
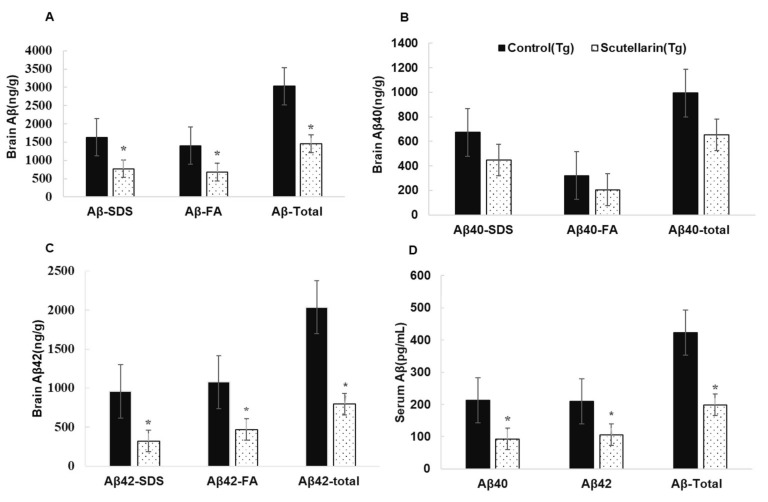
Effects of scutellarin consumption on Aβ levels in the brain of APP/PS1 transgenic mice. (**A**) Aβ peptide concentration in the brain and serum of each animal; (**B**) Comparison of total Aβ40, Aβ40-SDS, and Aβ40-FA; (**C**) Comparison of total Aβ42, Aβ42-SDS, and Aβ42-FA; (**D**) Comparison of total Aβ, Aβ40, and Aβ42 in serum, and denote * *p <* 0.05 versus APP/PS1control mice.

Following, the levels of soluble and insoluble Aβ1-40 and Aβ1-42 in the brain homogeneates were measured by ELISA kit. Figure 6B, C show scutellarin treatment group separately reduced soluble Aβ42 and insoluble Aβ42 66% and 56% (*p* < 0.05), reduced the levels of soluble Aβ40 37% and insoluble Aβ40 36% when comparing with untreated Tg mice (*p* < 0.05). ELISA assay also showed that the scutellarin treatment group had significantly decreased levels of total Aβ 52.2% and 53% in the brain homogenates and in the serum separately (*p* < 0.05, Figure 6A,D). These results indicate that scutellarin is effective at reducing both cerebral parenchymal deposition of the Aβ peptide levels in the brain and the plasma, possesses the ability clearance of Aβ from brain to periphery.

### 3.5. Scutellarin Decreases Reactive Gliosis and Proinflammatory Cytokines in APP/PS1mice

**Figure 7:**
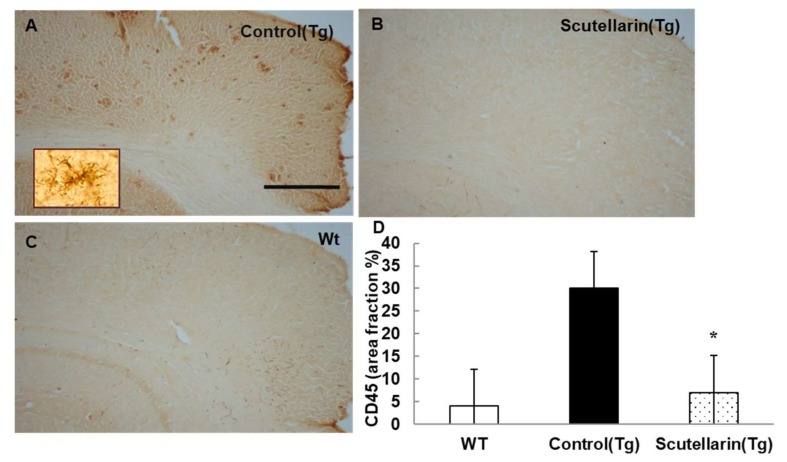
Scutellarin decreases microgliosis in the brain. (**A**) Microgliosis in neocortex of APP/PS1 control mice; (**B**) Scutellarin treated APP/PS1 transgenic mice; (**C**) Wild-type control mice; (**D**) Comparison of CD45 area fraction in neocortex and hippocampus among groups. Scutellarin significantly decreased the number of microgliosis in the brain of treated mice. * *p* < 0.05 versus APP/PS1 transgenic mice. Scalebar = 0.5 mm. Original magnification, 4×.

**Figure 8:**
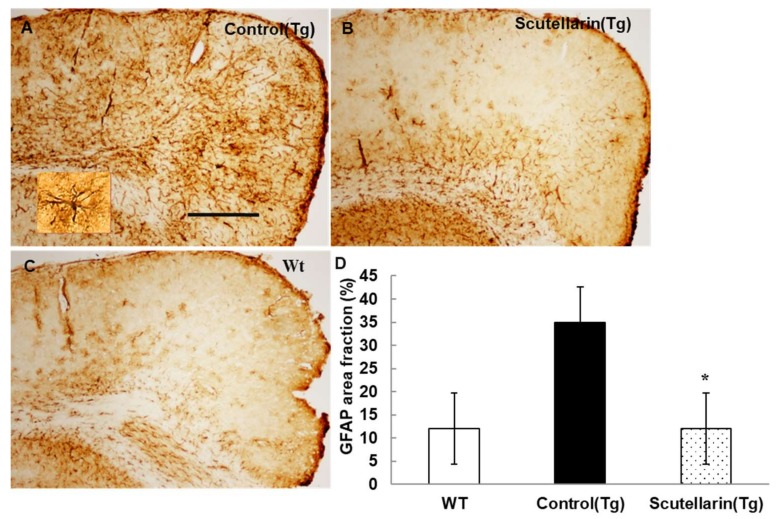
Scutellarin decreases astrogliosis in the brain. (**A**) Astrogliosis in neocortex of APP/PS1 control mice; (**B**) Scutellarin treated APP/PS1 transgenic mice; (**C**) Wild-type control mice; (**D**) Comparison of GFAP area fraction in neocortex and hippocampus among groups. Scutellarin significant decreased the number of astrogliosis in the brain of scutellarin treated mice. * *p* < 0.05 versus APP/PS1 transgenic mice. Scale bar = 0.5 mm. Original magnification, 4×.

**Figure 9:**
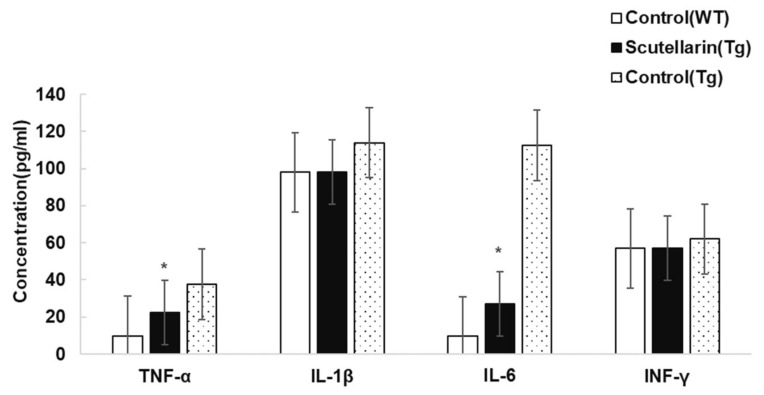
Effects of scutellarin on plasma levels of TNF-α, IL-1β, IL-6, and IFN-γ. Plasma levels of TNF-α and IL-6 were significantly decreased in the scutellarin treated APP/PS1 mice, * *p* < 0.05 versus APP/PS1 control mice. No significant differences are observed in plasma levels of IL-1β and IFN-γ among the groups, *p* > 0.05 versus APP/PS1 control mice.

Activated astrocytes and microglia facilitate Aβ clearance, but also mediate inflammation via cytokine production proinflammatory cytokines and immunostimulatory molecules [13]. Immunohistochemical staining for the microgliasis and astrogliosis in neocortical and hippocampal regions revealed that scutellarin treated group (Tg) had a significant lower level of microgliosis 76% and astrocytesis 66% compared to control Tg group (Figure 7 and Figure 8). In addition, plasma levels of proinflammatory cytokines TNF-α and IL-6 were significantly decreased 42% and 64% separately when compared with control Tg group mice, but concentrations of IL-1β and IFN-γ from plasma of scutellarin treated Tg mice did not show significant differences compared to the control Tg group (ANOVA, IL-β, F = 1.407, *p* > 0.05, IFN-γ, F = 0.297, *p* > 0.05, Figure 9).

## 4. Discussion

Aβ is believed to play a critical role in the AD pathology process. Aβ peptides subsequently aggregate from monomers to oligomers, protofibrils, and fibrils, inducing cognitive deficits and a series of the deleterious cascades that exacerbate neuronal injury [14], which consequently means they have been considered as a primary toxic species in AD [15]. Therefore, developing multi-target drugs to antagonize the aggregation of various amyloid proteins and interfere with the pathological process becomes an attractive therapeutic strategy.

Here, we used a series of techniques to investigate anti-amyloidogenic effects of scutellarin on AD pathology. In vitro, we determined the effects of scutellarin on the destabilization of Aβ fibrils by thioflavin T fluorescence spectroscopy and electron microscopy. Our results present that scutellarin dependently destabilized preformed Aβ; altered the Aβ aggregation pathway to yield non-toxic, unstructured Aβ aggregates; and displayed anti-aggregation effects on Aβ. In the cell culture experiment, cell growth was remarkably inhibited by Aβ oligomers treatment. However, scutellarin reduced the Aβ-induced cytotoxicity and exerted a neuroprotective effect in the SH-SY5Y cell culture model.

Through an in vivo study, the neuroprotective effects of a scutellarin diet have been tested in APP/PS1 transgenic mouse model. Our data showed that scutellarin significantly improved behavioral impairment, decreased Aβ levels in the brain and the serum, exhibited strong cleaning capacity in the Tg mice brain and the periphery, and did not cause side effects.

Brain inflammation is one of the hallmarks of AD and originates in the central nervous system. Microglia and astrocytes are arguably the major sources of cytokines in AD. Aβ complexes interact with microglial and astrocytic expressed pattern recognition receptors that initiate innate immunity [16]. This process leads to the production of toxic and inflammatory mediators such as hydrogen peroxide, nitric oxide, and cytokines—including interleukin (IL)-1β, IL-6, TNF-a, and IFN-γ etc.—that can recruit further microglia and astrocytes to the inflammatory site [17]. The passage of cytokines through the blood–brain barrier allows for the ability to peripherally measure them. In the present study, we found scutellarin reduced neuroinflammation including Aβ plaque-associated microgliosis and activated astrocytes, cytokines levels of TNF-α and IL-6, and exerted anti-inflammatory properties through downregulation of pro-inflammatory cytokine expression, thereby contributing to improved cognition and decreased Aβ concentrations.

Obviously, these beneficial effects can be attributed to its structure of phenol, which enables it to penetrate the blood–brain barrier and helps to re-establish the redox regulation of proteins, transcription factors, and signaling cascade; successfully protects neuronal cells from oxidative damage; and potentially alleviates neuroinflammation of AD [18,19]. In addition, the interaction of scutellarin with Aβ induces conformational and structural changes in Aβ oligomers, disrupts hydrophobic and π–π interactions of Aβ peptides and prevents Aβ aggregation and toxicity. In turn, the Aβ oligomer–scutellarin interaction results in delayed fibril formation, reduces amyloid plaque load, and attenuates behavior deficits in APP/PS1 mice. A published paper has discussed the molecular mechanism of scutellarin prevention of AD pathology [20]. Yuan et al. suggest that scutellarin regulates the activation of microglia via the Notch pathway and concurrently induces morphological and functional changes in activated microglia [21]. Xu et al. propose that scutellarin can effectively upregulate the synthesis and release of NGF, glial cell line-derived neurotrophic factor (GDNF), and brain-derived neurotrophic factor (BDNF) [22]. Chai et al. put forward that scutellarin can induce the expression of neurotrophin messenger RNAs and proteins through cyclic adenosine monophosphate response, element-binding protein (P-CREB), and p-Akt signaling and inhibit NO production in early stages of neuronal damage [23]. These conclusions indicate that scutellarin can slow down the progression of AD-like neuropathology via anti-inflammation, anti-oxidation, anti-amyloidogenic properties, consistent with ferulic acid and fisetin which have shown effects on improving behavioral impairment and Alzheimer-like pathology in transgenic mice [24,25]. Although scutellarin exerts multiple beneficial functions in mouse models of AD, the key question remains unanswered: can scutellarin’s beneficial actions on animal models be translated to the human condition? In addition, due to its poor solubility and weak oral absorption, the clinical use of scutellarin is limited [26]. In further research, we need to identify bioactive metabolites and dissect their targets in AD modification.

## 5. Conclusions

In conclusion, our studies have proven that scutellarin alleviates Alzheimer’s-like pathology and cognitive decline by reducing Aβ levels in the brain and plasma, decreasing Aβ plaque associated gliosis and levels of proinflammatory cytokines TNF-α and IL-6, and attenuating neuroinflammation. Our results suggest that scutellarin has potential for use as a therapeutic candidate for AD.

## References

[1] Querfurth H.W., Laferla F.M. (2010). Alzheimer’s disease. N. Engl. J. Med..

[2] Serrano-Pozo A., Frosch M.P., Masliah E., Hyman B.T. (2011). Neuropathological alterations in Alzheimer disease. Cold Spring Harb. Perspect. Med..

[3] Lakey-Beitia J., Berrocal R., Rao K.S., Durant A.A. (2015). Polyphenols as therapeutic molecules in Alzheimer’s disease through modulating amyloid pathways. Mol. Neurobiol..

[4] Huang D.Y., Li H.X., Zhang L.N., Lv Y.H., Cui H.D., Zheng J.H. (2010). Scutellarin promotes in vitro angiogenesis in human umbilical vein endothelial cells. Biochem. Biophys. Res. Commun..

[5] Wang S., Wang H., Guo H., Kang L., Gao X., Hu L. (2011). Neuroprotection of scutellarin is mediated by inhibition of microglial inflammatory activation. Neuroscience.

[6] Guo L.L., Guan Z.Z., Wang Y.L. (2011). Scutellarin protects against abeta-induced learning and memory deficits in rats: Involvement of nicotinic acetylcholine receptors and cholinesterase. Acta Pharmacol. Sin..

[7] Dahlgren K.N., Manelli A.M., Stine W.B., Baker L.K., Krafft G.A., LaDu M.J. (2002). Oligomeric and fibrillar species of amyloid-beta peptides differentially affect neuronal viability. J. Biol. Chem..

[8] Hudson S.A., Ecroyd H., Kee T.W., Carver J.A. (2009). The thioflavin t fluorescence assay for amyloid fibril detection can be biased by the presence of exogenous compounds. FEBS J..

[9] Jankowsky J.L., Slunt H.H., Ratovitski T., Jenkins N.A., Copeland N.G., Borchelt D.R. (2001). Co-expression of multiple transgenes in mouse cns: A comparison of strategies. Biomol. Eng..

[10] Lin L.L., Liu A.J., Liu J.G., Yu X.H., Qin L.P., Su D.F. (2007). Protective effects of scutellarin and breviscapine on brain and heart ischemia in rats. J. Cardiovasc. Pharmacol..

[11] Zeng Y.Q., Wang Y.J., Zhou X.F. (2014). Effects of (-)Epicatechin on the Pathology of APP/PS1 Transgenic Mice. Front Neurol..

[12] Wang Y.J., Pollard A., Zhong J.H., Dong X.Y., Wu X.B., Zhou H.D., Zhou X.F. (2009). Intramuscular delivery of a single chain antibody gene reduces brain abeta burden in a mouse model of Alzheimer’s disease. Neurobiol. Aging.

[13] Griffin W.S.T. (2006). Inflammation and neurodegenerative diseases. Am. J. Clin. Nutr..

[14] Ahmed M., Davis J., Aucoin D., Sato T., Ahuja S., Aimoto S., Elliott J.I., Van Nostrand W.E., Smith S.O. (2010). Structural conversion of neurotoxic amyloid-beta(1-42) oligomers to fibrils. Nat. Struct. Mol. Biol..

[15] Goure W.F., Krafft G.A., Jerecic J., Hefti F. (2014). Targeting the proper amyloid-beta neuronal toxins: A path forward for Alzheimer’s disease immunotherapeutics. Alzheimer Res. Ther..

[16] Minter M.R., Taylor J.M., Crack P.J. (2016). The contribution of neuroinflammation to amyloid toxicity in Alzheimer’s disease. J. Neurochem..

[17] Heneka M.T., Carson M.J., Khoury J.E., Landreth G.E., Brosseron F., Feinstein D.L. (2015). Neuroinflammation in Alzheimer’s disease. Lancet Neurol..

[18] Qu J., Wang Y., Luo G. (2001). Determination of scutellarin in erigeron breviscapus extract by liquid chromatography–tandem mass spectrometry. J. Chromatogr. A.

[19] Dajas F., Juan Andres A.C., Florencia A., Carolina E., Felicia R.M. (2013). Neuroprotective actions of flavones and flavonols: Mechanisms and relationship to flavonoid structural features. Cent. Nerv. Syst. Agents Med. Chem..

[20] Guo L.L., Guan Z.Z., Huang Y., Wang Y.L., Shi J.S. (2013). The neurotoxicity of beta-amyloid peptide toward rat brain is associated with enhanced oxidative stress, inflammation and apoptosis, all of which can be attenuated by scutellarin. Exp. Toxicol. Pathol..

[21] Yuan Y.R.P., Kan E.M. (2015). Scutellarin regulates the notch pathway and affects the migration and morphological transformation of activated microglia in experimentally induced cerebral ischemia in rats and in activated bv-2 microglia. J. Neuroinflamm..

[22] Xu S.L., Bi C.W., Choi R.C., Zhu K.Y., Miernisha A., Dong T.T., Tsim K.W. (2013). Flavonoids induce the synthesis and secretion of neurotrophic factors in cultured rat astrocytes: A signaling response mediated by estrogen receptor. Evid. Based Complement. Altern. Med..

[23] Chai L., Guo H., Li H., Wang S., Wang Y.L., Shi F., Hu L.M., Liu Y., Adah D. (2013). Scutellarin and caffeic acid ester fraction, active components of dengzhanxixin injection, upregulate neurotrophins synthesis and release in hypoxia/reoxygenation rat astrocytes. J. Ethnopharmacol..

[24] Mori T., Koyama N., Guillot-Sestier M.V., Tan J., Town T. (2013). Ferulic acid is a nutraceutical beta-secretase modulator that improves behavioral impairment and Alzheimer-like pathology in transgenic mice. PLoS ONE.

[25] Currais A., Prior M., Dargusch R., Armando A., Ehren J., Schubert D., Quehenberger O., Maher P. (2014). Modulation of p25 and inflammatory pathways by fisetin maintains cognitive function in Alzheimer’s disease transgenic mice. Aging Cell.

[26] Chen X., Cui L., Duan X., Ma B., Zhong D. (2006). Pharmacokinetics and metabolism of the flavonoid scutellarin in humans after a single oral administration. Drug Metab. Dispos..

